# Associations Between Vitamin D Status and Clinical Presentation Among French Inpatients With Substance Use Disorders

**DOI:** 10.1111/adb.70110

**Published:** 2026-01-08

**Authors:** Luca Pavirani, Bibi Aliya Seelarbokus, Léa Marinelli, Pauline Desnavailles, Sylvie Berthoz, Melina Fatseas

**Affiliations:** ^1^ Univ. Bordeaux, INCIA CNRS‐UMR 5287 Bordeaux France; ^2^ CHU Bordeaux Bordeaux France; ^3^ CH Charles Perrens Bordeaux France; ^4^ Univ. Bordeaux LabPsy Bordeaux France; ^5^ Department of Psychiatry for Adolescents and Young Adults Institut Mutualiste Montsouris Paris France

**Keywords:** calcium, craving for drugs, craving for food, C‐reactive protein, micronutrients, substance use disorders

## Abstract

**Background:**

Numerous studies have demonstrated that vitamin D plays a crucial role in modulating dopaminergic pathways, suggesting a potential role in the pathophysiology of addictive disorders. However, among individuals with substance use disorders (SUDs), the link between vitamin D and addiction‐related symptoms has not been sufficiently studied.

**Objective:**

This study aims to explore among inpatients treated for various types of SUDs differences in addiction‐related, psychopathological and biological characteristics based on vitamin D status.

**Methods:**

The sample included 260 participants hospitalized in a French addiction treatment unit. Vitamin D concentration and other biomarkers, SUDs severity and other psychiatric disorders, cognitive functioning and impulsivity were collected at hospital admission (T0). Perceived negative affectivity and craving (for the main substance and food) were collected at T0 and hospital discharge (T1).

**Results:**

About 89.3% of the participants had a vitamin D deficiency as defined by French recommendations (< 30 ng/mL). Using univariate between‐group analyses, vitamin D deficiency was associated with higher body weight and lower calcium plasma levels at T0, as well as increased craving intensity for both the substance at the origin of the treatment and food at T1. Multivariate regression analyses showed no significant associations between vitamin D or calcium levels and craving intensities at T1.

**Conclusion:**

This study confirms the high prevalence of vitamin D deficiency among inpatients treated for SUDs. Although preliminary, our findings highlight the importance of assessing vitamin D levels in SUDs. They call for further research on its role in relapse vulnerability and the potential benefits of its supplementation during drug withdrawal treatment.

## Graphical abstract

Nine out of ten inpatients treated for various types of substance use disorders had a Vitamin D deficiency. Vitamin D deficiency was associated with lower calcium plasma levels, higher BMI and craving levels for both substance and food. Vitamin D supplementation should be part of the treatment regimen of people with substance use disorders.

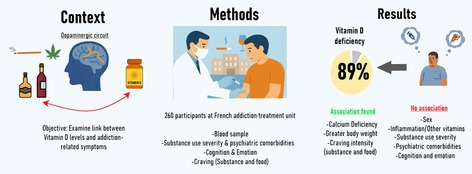

## 1 Introduction

Vitamin D status has been the focus of growing interest in the past decade, with increasing evidence implicating serum 5‐hydroxyvitamin D (25(OH)D) in neuroprotection and neurodevelopment, beyond its well‐known effects on calcium and phosphate metabolism [1]. After the discovery and validation of vitamin D receptors (VDRs) in diverse brain regions and their interaction with the transcriptional machinery inside target cells [2], an increasing number of experimental animal studies have demonstrated direct interactions between vitamin D and neurotrophic factors such as nerve growth factor (NGF) and glial cell line‐derived neurotrophic factor (GDNF), both of which are crucial for cell proliferation and neurodevelopment [3].

In animal models, vitamin D administration has been shown to enhance neuroprotection, while VDR knockout mice exhibited behavioural disorders [3]. Mechanistically, as vitamin D status may affect dopamine synthesis either directly or indirectly, the neuroprotective effects of vitamin D have been purported to involve dopaminergic pathways [2, 3, 4]. Vitamin D administration has been found to reduce dopamine depletion in methamphetamine‐treated mice [5, 6], besides exhibiting a protective effect on dopaminergic neurons from neurotoxic damage [2]. In this context, it has been hypothesized that vitamin D deficiency may play a crucial role in the physiopathology of psychiatric disorders, notably in addictive disorders [4], by disrupting dopaminergic pathways that are critical in the development and maintenance of addictions [7, 8].

In humans, vitamin D deficiency has been associated not only with a wide range of autoimmune and neurodegenerative diseases but also with psychiatric disorders, including addiction, depression and schizophrenia, with more severe symptoms being associated with poorer vitamin D status [3, 9, 10]. Regarding addictive disorders, although data on vitamin D status in people with substance use disorders (SUDs) compared to healthy controls remain contradictory [11, 12, 13], several methodologically robust epidemiological studies document a high prevalence of vitamin D deficiency in SUD populations, with preliminary data suggesting a potential beneficial effect of vitamin D on psychiatric and substance outcomes [14, 15]. However, in contrast to other psychiatric diseases such as depression or schizophrenia, the potential involvement of vitamin D deficiency in the development and maintenance of SUDs has, until now, been studied insufficiently in clinical samples; particularly, data investigating the link between vitamin D status and addiction‐related symptoms and severity warrant further investigation [15].

The plasma concentration of 25(OH)D, the active metabolite of 1,25‐dihydroxyvitamin D [1,25(OH)_2_D], is the usually recommended indicator of vitamin D status, reflecting vitamin D supply and usage over time [16]. Although there is no international consensus to define optimal serum levels, a concentration ≥ 30 ng/mL (≥ 75 nmol/L) of 25(OH)D has been considered optimal [17], with levels < 30 ng/mL indicating deficiency [18]. In SUD populations, several studies have shown higher rates of vitamin D deficiency compared to the general population, with significantly lower 25(OH)D plasma levels [14, 19, 20, 21, 22]. For instance, Bemanian et al. found that 57% of 666 substance‐dependent Norwegian outpatients exhibited vitamin D deficiency, compared to less than 40% in the general population [14]. Importantly, besides typical confounding factors which are known to influence vitamin D levels in the general population such as sex, skin pigmentation, body mass index (BMI) as well as sunlight exposure, in SUD populations, poor nutritional status, micronutrient deficiencies, lifestyle, malabsorption and hepatic damage [23, 24] may represent the most important factors contributing to decreased vitamin D levels. Furthermore, depression—a highly comorbid condition among individuals with SUDs—has been associated with a higher risk of hypovitaminosis D [25].

Despite the association reported between vitamin D status and addictive disorders, clinical studies investigating symptoms related to SUDs remain scarce and often have poor consideration of confounding variables [15]. A systematic review [13] highlighted controversial findings on the association between vitamin D status and alcohol use, and the discrepancies between the studies were partly explained by methodological limitations. Few studies have examined the potential relationship of vitamin D status with several features of the clinical profile in SUD samples. Neupane et al. [26] found among alcohol use disorder (AUD) inpatients that lower vitamin D levels were associated with higher scores on the alcohol use disorders identification test (AUDIT) questionnaire and their relapse history. More recently, longitudinal data from a large cohort of opioid use disorder (OUD) outpatients indicated an association between poor vitamin D status and greater cannabis use at baseline but with no association with substance outcomes at follow‐up [14]. Additionally, a retrospective study among 5446 individuals undergoing surgical procedures showed that preoperative vitamin D deficiency was associated with postoperative increased opioid duration/dose as well as incident OUD [27].

Given the high frequency of vitamin D deficiency in SUD samples and its potential impact on substance use patterns, there is a need to further examine whether poor vitamin D status may be linked to substance use symptoms and severity, controlling for other confounding factors such as depression symptoms or nutritional status. Furthermore, whereas higher craving intensity and cue‐reactivity have been recently found to be associated with lower calcium levels among AUD inpatients [20, 28, 29], the extent to which vitamin D levels may be associated with craving intensity, a core feature of addictive disorders and a predictor of substance use and relapse, has not yet been investigated. Considering that the mechanism of action between vitamin D, calcium and SUD‐related symptoms such as substance craving and addiction severity remains to be elucidated, but based on the literature mentioned above, it may be that vitamin D deficiency interacts with SUD features, especially impulsivity and craving intensity, through its role in dopaminergic transmission, leading to increased substance use and relapse.

To advance scientific knowledge in this research field, the objective of the present study was to explore in a sample of inpatients treated for different types of substance addictions the association between vitamin D status and (i) their clinical and biological profile at hospital admission, as well as (ii) their levels of craving and emotional distress at hospital discharge.

## 2 Methods

### 2.1 Participants

This study was part of a larger protocol (the SUED protocol: substance use and eating disorders: food craving and addiction transfer) that consecutively recruited inpatients and outpatients who sought treatment for any type of addiction (substance and non‐substance) and eating disorders at the Bordeaux University Hospital (France). The SUED protocol was approved by the Institutional Human Research Committee (ClinicalTrials.gov NCT05315635). The research was conducted in accordance with the Helsinki Declaration as revised in 1989. All participants provided their written informed consent.

For the present study, the specific inclusion criteria were as follows: having a current diagnosis of SUD (DSM‐5 criteria), being ≥ 18 years old, and hospitalized between January 2020 and March 2024 for at least 3‐week treatment programme within the specialized addiction unit.

### 2.2 Procedure

Upon admission (T0), participants received a comprehensive baseline clinical assessment of substance use/addictive disorders and associated symptoms, psychiatric comorbidities, cognitive status, impulsivity, as well as emotional distress, and venous blood samples were performed for biological values. Levels of emotional distress and craving were again collected at hospital discharge (T1), which occurred 21 or 28 days after admission.

### 2.3 Measures

#### 2.3.1 Socio‐Demographic and Medical Characteristics

Age, sex and the level of education were collected based on medical records. BMI (kg/m^2^) as well as nutritional status were determined by a clinician upon admission. For nutritional status, the presence of malnutrition (yes/no) was determined using the standard criteria from the French National guidelines [30].

#### 2.3.2 Biological Data

Determination of plasma 25(OH)D concentration was performed by the medical laboratory of the Bordeaux University Hospital using the Architect 25‐OH Vitamin D assay (chemiluminescent microparticle immunoassay) on the Abbott Architect instrument. Following the French guidelines, we defined 25(OH)D levels ≥ 30 ng/mL as optimal and 25(OH)D levels < 30 ng/mL as deficient.

Serum concentrations of calcium, vitamin B1, B6, B9, B12 and C‐Reactive Protein levels (CRP) were also collected.

#### 2.3.3 Addiction‐Related Characteristics

- The main substance that was at the origin of the treatment and smoking status was collected based on medical records. According to the availability of the required resources, SUD diagnosis and severity were further assessed with the Mini International Neuropsychiatric Interview (MINI) (MINI 7.0.2 (8/8/16) DSM‐5 version from Sheehan D.V; French version by MAPI Research Trust). A diagnosis of poly‐addiction was defined as having at least two current SUDs according to the MINI, but excluding tobacco addiction, as it is very common among individuals treated for other substance addictions than tobacco.
- The mean craving intensities (mean intensity over the seven previous days and current intensity) for the main substance as well as for food were assessed with a visual analogue scale ranging from 0 (no craving) to 10 (extreme craving) at T0 and T1.

#### 2.3.4 Psychiatric Comorbidities and Psychopathological Characteristics

- The MINI was also used to investigate psychiatric comorbidities for the following diagnoses (current and past): mood disorders (major depressive episode, bipolar disorder), psychotic symptoms, adult attention‐deficit/hyperactivity disorder (ADHD) and antisocial personality.
- Perceived levels of emotional distress were investigated with the hospital anxiety and depression scale (HAD) [31], a 14‐item self‐report questionnaire that assesses the level of anxious (seven items) and depressive (seven items) symptoms over the past week using a 4‐point Likert scale rating from 0 to 3. Higher scores indicate higher levels of symptoms.
- The short form of the UPPS Impulsive Behaviour scale UPPS‐P [32] was used to evaluate in 20 items five different impulsivity facets (four items each): negative urgency, positive urgency, lack of premeditation, lack of perseverance and sensation seeking. Each item is rated on a Likert scale ranging from 1 (*I agree strongly*) to 4 (*I disagree strongly*), where higher scores indicate higher propensity to behave impulsively.

#### 2.3.5 Cognitive Status

The montreal cognitive assessment (MoCA) is a short test that assesses a range of cognitive abilities, including memory, visuospatial abilities, executive function, attention and concentration, language and orientation. A cut‐off of ≤ 25 has been recommended to detect mild cognitive impairment. It has been validated in SUD populations [33].

### 2.4 Statistical Analyses

Continuous variables were described by means and standard deviations, categorical variables as numbers and percentages.

Vitamin D groups (Optimal: ≥ 30 ng/mL; Deficient: < 30 ng/mL) were compared using chi‐square tests or Fisher's nonparametric test where appropriate for the qualitative variables and using Student's *t*‐tests or Mann–Whitney tests as appropriate. In addition, to further explore a potential prospective link between vitamin D or calcium and craving and due to the extreme imbalance in sample size between the Vitamin D groups, step‐wise regression analyses using vitamin D or calcium levels as continuous predictors were performed. Variables with *p* ≤ 0.20 in univariate analyses were entered into the regressions to avoid excluding potentially relevant predictors.

Missing data were not imputed and are reported in the tables.

Due to the exploratory nature of our study, differences were considered statistically significant when the *p*‐value was ≤ 0.05 with no correction for multiple comparisons.

Analyses were conducted using Jamovi Software (version 2.4.14.0).

## 3 Results

### 3.1 Description of the Study Sample

Between January 2020 and March 2024, 539 inpatients were eligible, of whom 289 (53.6%) could be approached and asked to participate in the study; all agreed to complete the evaluations. Among them, vitamin D concentration was available for 260 participants, who had an average stay of 25 days.

Table 1 presents the sociodemographic, clinical and biological characteristics of the 260 study participants. They were mainly males (70.8%; 184/260), had a mean age of 44.4 years (SD = 11.5), a mean BMI of 24.6 kg/m^2^ (SD = 5.7) and with 20.0% (52/260) meeting criteria for malnutrition. Among the participants who completed the MoCA, 47.8% (107/224) had a score ≤ 25.

**TABLE 1:** Descriptive statistics of the overall (*N* = 260) study population.

Characteristics	Mean ± SD or number (%)
Sociodemographic data	
Age (years)	44.4 ± 11.5
Sex (male)	184 (70.8)
Level of education	
High school degree	130 (50.3)
Bachelor's degree	60 (21.1)
University	80 (28.2)
Medical characteristics	
BMI, kg/m^2^ (*n* = 259)	24.6 ± 5.7
Malnutrition (yes) (*n* = 253)	52 (20.0)
MoCA (*n* = 224)	25.1 ± 3.4
Vitamin D (ng/mL) (*n* = 260)	16.8 ± 11.4
Vitamin B1 (nmol/L) (*n* = 244)	187.8 ± 78.7
Vitamin B6 (nmol/L) (*n* = 242)	427.8 ± 1355.7
Vitamin B9 (ng/mL) (*n* = 220)	30.3 ± 339.0
Vitamin B12 (pg/mL) (*n* = 223)	447.7 ± 238.8
Calcium (mmol/L) (*n* = 240)	2.3 ± 0.1
CRP (mg/L) (*n* = 248)	7.0 ± 12.9
Addiction characteristics	
Current smokers (*n* = 260)	195 (75.0)
Substance at the origin of the treatment (*n* = 260)	
Alcohol	191 (73.5)
Cannabis	10 (3.8)
Cocaine and other stimulants	39 (15.0)
Opiates	13 (5.0)
Benzodiazepines	4 (1.5)
Other	3 (1.2)
Current substance use disorders (MINI)	
Alcohol use disorder (AUD) (*n* = 204)	171 (83.8)
Mild	9 (4.4)
Moderate	16 (7.8)
Severe	146 (71.6)
Cannabis use disorder (CUD) (*n* = 200)	40 (20)
Mild	5 (2.5)
Moderate	14 (7.0)
Severe	21 (10.5)
Cocaine and other stimulants use disorder (COD) (*n* = 200)	54 (27)
Mild	4 (2.0)
Moderate	3 (1.5)
Severe	47 (23.5)
Opiate use disorder (OUD) (*n* = 201)	15 (7.5)
Mild	0 (0.0)
Moderate	0 (0.0)
Severe	15 (7.5)
Benzodiazepine use disorder (BUD) (*n* = 200)	11 (5.5)
Mild	0 (0.0)
Moderate	3 (1.5)
Severe	8 (4.0)
Other use disorder (*n* = 201)	8 (4.0)
Mild	3 (1.5)
Moderate	2 (1.0)
Severe	3 (1.5)
Polyaddiction^a^ (yes) (*n* = 200)	85 (42.5)
Main substance craving intensity	
T0‐craving over the previous 7 days (*n* = 230)	4.4 ± 3.4
T0‐current craving (*n* = 230)	2.8 ± 3.0
T1‐craving over the previous 7 days (*n* = 132)	2.4 ± 2.6
T1‐current craving (*n* = 131)	1.5 ± 2.3
Food craving intensity	
T0‐craving over the previous 7 days (*n* = 251)	4.1 ± 3.0
T0‐current craving (*n* = 254)	4.0 ± 3.1
T1‐craving over the previous 7 days (*n* = 157)	3.4 ± 2.8
T1‐current craving (*n* = 158)	3.2 ± 2.8
Psychiatric comorbidity (MINI current or past)	
Major depressive episode (MDE) (*n* = 207)	141 (68.1)
Bipolar disorder (I or II) (BD) (*n* = 205)	74 (36.1)
Adult attention deficit hyperactivity disorder (ADHD) (*n* = 192)	62 (32.3)
Psychotic symptoms (*n* = 201)	9 (4.5)
Antisocial personality disorder (*n* = 178)	34 (19.1)
Emotional distress	
HAD anxiety	
T0 (*n* = 254)	10.3 ± 4.4
T1 (*n* = 158)	8.5 ± 4.4
HAD depression	
T0 (*n* = 254)	8.2 ± 3.9
T1 (*n* = 158)	6.0 ± 3.9
Impulsivity	
UPPS‐negative urgency (*n* = 209)	10.8 ± 2.3
UPPS‐positive urgency (*n* = 209)	10.9 ± 2.6
UPPS‐premeditation (lack) (*n* = 207)	8.6 ± 2.6
UPPS‐perseveration (lack) (*n* = 206)	8.5 ± 3.0
UPPS‐sensation seeking (*n* = 209)	10.6 ± 2.9

*Note:* %: percentage.

Abbbreviations: ADHD, attention‐deficit/hyperactivity disorder; AUD, alcohol use disorder; BD, bipolar disorder; BMI, body mass index; BUD, benzodiazepine use disorder; COD, cocaine and other stimulants use disorder; CUD, cannabis use disorder; CRP, C‐reactive protein; HAD, hospital anxiety and depression scale, MDE, major depressive episode; MINI, mini international neuropsychiatric interview; MoCA, montreal cognitive assessment, OUD, opiate use disorder; PD, personality disorder; SD, standard deviation, UPPS, impulsive behavior scale short form.

^a^ Excluding tobacco.

Mean plasma vitamin D concentration of the overall sample was 16.8 ng/mL (SD = 11.4), ranging from less than 2.4–67.1 ng/mL. Overall, 89.3% (232/260) of the participants were considered as deficient (25(OH)D < 30 ng/mL), including 20.4% (53/260) with a concentration between ≥ 20 and < 30 ng/mL, 34.6% (90/260) between ≥ 10 and < 20 ng/mL and 34.2% (89/260) with a concentration < 10 ng/mL.

Regarding substance use, 75.0% (195/260) of the overall sample were active smokers. The main SUD at the origin of the treatment was alcohol (73.5%, 191/260), followed by cocaine and other stimulants (15.0%, 39/260), opiates (5.0%, 13/260), cannabis (3.8%, 10/260), benzodiazepines and related substances (1.5%, 4/260) and 1.2% (3/260) with other substances addiction.

For the subsample evaluated using the MINI, 83.8% (171/204) of the respondents met DSM‐5 criteria for a current AUD, 27% (54/200) for a current cocaine and other stimulant use disorder, 20% (40/200) for a current cannabis use disorder, 7.5% (15/201) for a current opioid use disorder, 5.5% (11/200) for a current benzodiazepine use disorder and 4% (8/201) for other substances addiction. Based on these diagnoses, and excluding tobacco, 42.5% of this subsample of participants presented with polyaddiction.

### 3.2 Comparisons by Vitamin D Status

Tables 2, 3, 4 show the descriptive statistics and comparison of the two vitamin D groups according (232 < 30 ng/mL; 28 ≥ 30 ng/mL).

**TABLE 2:** Group comparisons by vitamin D status for socio‐demographic and medical characteristics.

	Deficit (*N* = 232)	Optimal (*N* = 28)	*p*	Effect size
Sociodemographic characteristics				
Age (years) (*n* = 232 vs. 28)	44.3 ± 11.3	45 ± 13.6	0.775	−0.057
Sex (male) (*n* = 232 vs. 28)	168 (72.4)	16 (57.1)	0.093	0.104
Level of education (*n* = 228 vs. 27)			0.23	0.174
High school degree	122 (53.5)	8 (29.6)		
Bachelor's degree	46 (20.2)	9 (33.3)		
University	60 (26.4)	10 (37)		
Medical characteristics				
BMI (*n* = 231 vs. 28)	24.8 ± 5.7	22.7 ± 4.9	**0.04**	0.237
Malnutrition (*n* = 225 vs. 28)	45 (20)	7 (25)	0.537	0.039
MoCA (n =199 vs. 25)	25.1 ± 3.6	25.3 ± 2.9	0.867	0.021
Vitamin B1 (*n* = 217 vs. 27)	187.4 ± 78.0	192 ± 85.9	0.677	0.05
Vitamin B6 (*n* = 217 vs. 25)	397.4±1350.8	714.8 ± 1394.0	0.096	0.207
Vitamin B9 (*n* = 195 vs. 25)	33.5 ± 359.2	8.2 ± 7.8	0.507	0.083
Vitamin B12 (*n* = 197 vs. 26)	449.8 ± 244.4	431.9 ± 192.5	0.806	0.03
Calcium (*n* = 214 vs. 26)	2.3 ± 0.1	2.4 ± 0.1	**0.046**	−0.424
CRP (*n* = 221 vs. 27)	7 ± 13.2	7 ± 10.6	0.61	0.061

*Note:* Values in bold are significant *p*‐values.

Abbreviations: BMI, body mass index; CRP: C‐reactive protein; MoCA, Montreal cognitive assessment.

**TABLE 3:** Group comparisons by vitamin D status for addictive and psychopathological characteristics.

Addiction characteristics	Deficit (*N* = 232)	Optimal (*N* = 28)	*p*	Effect size
	*N* (%)	*N* (%)		
Current smokers	175 (75.4)	20 (71.4)	0.647	0.033
Substance at the origin of the treatment				
Alcohol	172 (74)	19 (67.9)	0.477	0.044
Cannabis	10 (4.3)	0 (0)	0.607	0.07
Cocaine or stimulants	34 (14.7)	5 (17.9)	0.585	0.028
Opiates	12 (5.2)	1 (3.6)	1	0.023
Benzodiazepine	2 (0.9)	2 (7.1)	0.059	0.158
Other	2 (0.9)	1 (3.6)	0.291	0.079
Main substance craving intensity	M ± SD	M ± SD	*p*	Effect size
T0: over the previous 7 days (*n* = 205 vs. 25)	4.4 ± 3.4	3.9 ± 3.4	0.462	0.091
T0: current (*n* = 206 vs. 25)	2.8 ± 3.0	2.1 ± 2.7	0.228	0.146
T1: over the previous 7 days (*n* = 120 vs. 11)	2.6 ± 2.6	0.9 ± 1.7	**0.021**	0.411
T1: current (*n* = 120 vs. 11)	1.6 ± 2.4	0.1 ± 0.3	**0.016**	0.398
Food craving intensity	M ± SD	M ± SD	*p*	Effect size
T0: over the previous 7 days (*n* = 223 vs. 28)	4.1 ± 3.0	3.7 ± 2.9	0.456	0.087
T0: current (*n* = 227 vs. 28)	4.0 ± 3.1	3.6 ± 3.1	0.473	0.084
T1: over the previous 7 days (*n* = 141 vs. 15)	3.7 ± 2.8	1.5 ± 1.8	**0.003**	0.468
T1‐ current (*n* = 141 vs. 17)	3.4 ± 2.8	1.9 ± 2.1	**0.043**	0.299
Emotional distress	M ± SD	M ± SD	*p*	Effect size
HAD anxiety				
T0 (*n* = 227 vs. 27)	10.3 ± 4.3	10.5 ± 4.9	0.853	‐0.039
T1 (*n* = 158: 142 vs. 16)	8.7 ± 4.4	6.8 ± 4.1	0.103	0.432
HAD depression				
T0 (*n* = 227 vs. 27)	8.1 ± 3.9	8.7 ± 4.4	0.473	‐0.149
T1 (*n* = 142 vs. 16)	6.2 ± 3.9	4.3 ± 3.0	0.073	0.274
Impulsivity	M ± SD	M ± SD	*p*	Effect size
UPPS‐negative urgency (*n* = 181 vs. 24)	10.8 ± 2.4	10.8 ± 1.9	0.782	0.035
UPPS‐positive urgency (*n* = 183 vs. 24)	10.9 ± 2.7	11.1 ± 2.6	0.923	0.013
UPPS‐premeditation (lack) (*n* = 180 vs. 21)	8.6 ± 2.6	8.6 ± 2.3	0.867	0.023
UPPS‐perseveration (lack) (*n* = 181 vs. 24)	8.5 ± 3.0	8.8 ± 2.8	0.652	0.058
UPPS‐sensation seeking (*n* = 180 vs. 24)	10.6 ± 2.9	10.8 ± 3.5	0.898	0.017

*Note:* %: percentage. Values in bold are significant *p*‐values.

Abbreviations: HAD, hospital anxiety and depression scale; M, mean; N, number; SD, standard deviation; UPPS, impulsive behavior scale short form.

**TABLE 4:** Group comparisons by vitamin D status for addiction‐related and other psychiatric diagnoses according to the MINI.

Addiction characteristics	Deficit (*N* = 232)	Optimal (*N* = 28)	*p*	Effect size
	*N* (%)	*N* (%)		
Polyaddiction^a^ (*n* = 177 vs. 23)	75 (42.4)	10 (43.5)	0.92	0.007
Current SUD				
AUD (*n* = 180 vs. 24)			0.119	0.17
Mild	9 (5.0)	0 (0)		
Moderate	12 (6.7)	4 (16.7)		
Severe	132 (73.3)	14 (58.3)		
CUD (*n* = 177 vs. 23)			0.378	0.145
Mild	5 (2.8)	0 (0)		
Moderate	14 (7.9)	0 (0)		
Severe	20 (11.3)	1 (4.3)		
COD (*n* = 177 vs. 23)			0.916	0.07
Mild	4 (2.3)	0 (0)		
Moderate	3 (1.7)	0 (0)		
Severe	41 (23.2)	6 (26.1)		
OUD (*n* = 177 vs. 24)			0.388	0.076
Mild	0 (0)	0 (0)		
Moderate	0 (0)	0 (0)		
Severe	12 (6.7)	3 (13.0)		
BUD (*n* = 177 vs. 23)			0.132	0.122
Mild	0 (0)	0 (0)		
Moderate	2 (1.1)	1 (4.3)		
Severe	6 (3.4)	2 (8.7)		
Other (*n* = 178 vs. 23)			0.229	0.125
Mild	2 (1.1)	1 (4.3)		
Moderate	2 (1.1)	0 (0)		
Severe	2 (1.1)	1 (4.3)		
Psychiatric comorbidity (current or past)				
MDE (*n* = 182 vs. 25)	120 (65.9)	21 (84)	0.069	0.126
BD (I or II) (*n* = 180 vs. 25)	66 (36.7)	8 (32)	0.649	0.032
Adult ADHD (*n* = 169 vs. 23)	55 (32.5)	7 (30.4)	0.83	0.015
Psychotic symptoms (*n* = 177 vs. 24)	8 (4.5)	1 (4.2)	1	0.006
Antisocial PD (*n* = 156 vs. 22)	30 (19.2)	4 (18.2)	1	0.009

*Note:* %: percentage.

Abbreviations: ADHD, attention‐deficit/hyperactivity disorder; AUD, alcohol use disorder; BD, bipolar disorder; BUD, benzodiazepine use disorder; COD, cocaine and other stimulants use disorder; CUD, cannabis use disorder; MDD, major depressive episode; MINI, mini international neuropsychiatric interview; N, number, OUD, opioid use disorder; PD, personality disorder; SUD, substance use disorder.

^a^ Excluding tobacco.

There was a significant effect of group for BMI and calcium plasma levels, with significantly higher BMIs (*p* = 0.040; effect size = 0.237) and lower calcium levels (*p* = 0.046; effect size = −0.424) in the vitamin D‐deficient group. No other significant differences were found on any of the other variables measured at T0.

At T1, there was a significant effect of group for the levels of craving, with significantly higher mean craving intensity over the previous seven days and current craving intensity for both the substance at the origin of the treatment (respectively: *p* = 0.021, effect size = 0.411; *p* = 0.016, effect size = 0.398) and for food (*p* = 0.003, effect size = 0.468; *p* = 0.043, effect size = 0.299).

Regression analyses using vitamin D or calcium levels as continuous predictors of the craving intensity metrics at T1 showed no significant prospective associations (see Supporting Information).

## 4 Discussion

To the best of our knowledge, this is the first study that examined the association between vitamin D status and a range of clinical as well as biological factors in a hospital‐based cohort of people with various types of SUDs. Our results are in line with existing data demonstrating a high prevalence of vitamin D deficiency in SUD populations.

As expected, our study documented an overall poor vitamin D status among a sample of French inpatients undergoing treatment for severe SUDs, predominantly AUD, with an overall mean vitamin D plasma level of 16.8 ng/mL. The prevalence of people with a vitamin D deficiency among our study population is twice as high as that from the general French population reported in the ESTEBAN study (68.8% vs. 34.5% for vitD < 20 ng/mL) [18]. In particular, the prevalence of severe deficiency (< 10 ng/mL) in our cohort was five times higher compared to that observed in this study (34.2% vs. 6.5%). Regarding the literature in SUDs, the present findings are in line with previous studies, although the rate of severe deficiency appeared slightly higher. A study conducted in Nepal among 174 AUD inpatients (including 29 with comorbid CUD or BUD) reported a mean plasma level of 17.6 ng/mL, with 64% of the sample having a concentration < 20 ng/mL (68.8% in our study), though with 16% having a severe deficiency [26]. The discrepancies could be due to the prevalence of people with polyaddiction (only 16.7% in their study). Similarly, in a study in Norway involving 666 SUD outpatients, including 89% undergoing opiate agonist treatment [14], the baseline mean plasma level was 18 ng/mL, with 57% of the sample having a concentration < 20 ng/mL and 19% with a concentration < 10 ng/mL.

In the literature, being female, having a lower level of education and higher BMI, malabsorption, poor nutritional status and co‐occurring depression are factors that have all been linked with low serum 25(OH)D levels [34, 35]. In the present study, only a higher BMI was associated with vitamin D deficiency.

Though not expected, no association was found between Vitamin D status and depression, anxiety, as well as any dimensions of impulsivity. Impulsivity is a core component of addictive patterns that has previously been associated with vitamin D deficiency, both in preclinical [36] and clinical settings in two studies in people with an eating disorder [37, 38]. In SUD populations, only one study assessed this relationship and found an association between impulsivity and genetic variation of the VDR gene in AUD male patients [39]. Additionally, available findings on the link between vitamin D and mood status among SUD patients are inconsistent, thus warranting further investigation. While no association was found among people with AUD [26], vitamin D levels were positively correlated to anxiety and depression scores in 500 patients under methadone treatment [40], and beneficial effects of vitamin D supplementation on depression scores were reported in a randomized controlled trial among 68 patients with maintenance methadone treatment [41].

Concerning the relationships between vitamin D status and symptoms and/or severity of addictive disorders, vitamin D deficiency was associated with a higher intensity of craving at discharge, both for the substance at the origin of the treatment and for food. Of note, craving is considered a key feature of addictive disorders and a predictor of relapse, with a direct correlation observed between craving intensity and substance use when assessed in close temporal proximity, as used in daily‐life studies, and when patients are followed for long periods [42, 43]. As no effect of vitamin D status was found for the craving intensities at admission, it could be interesting to examine in future studies if SUD patients with a vitamin D deficiency potentially exhibit worse craving evolution in the course of withdrawal and thereby a higher relapse risk.

While data related to the association between SUD symptoms and vitamin D status in SUD populations remain scarce, the existing literature mainly focuses on patients treated for opiate use disorder (OUD) [14, 40, 44] or AUD [20, 26, 28], and there is less attention given to other types of addictions. In AUD inpatients, one observational study evidenced an association between poor vitamin D status, higher AUDIT scores and history of relapse [26] but no link with the amount or frequency of alcohol intake. In a study by Benamian et al. [14], there was no association between vitamin D status and frequency of substance use among opiate agonist treatment outpatients, except for cannabis. Only two studies investigated the link between vitamin D/calcium plasma levels and craving, both involving inpatients with AUD. Specifically, in one cohort study among 47 inpatients, no association was found between vitamin D and alcohol craving, while it was the case for calcium plasma level [20]. In the present study, the multivariate analyses showed no significant prospective association between levels of vitamin D and craving intensities (neither for the substance nor for food) and only as a trend for the association between levels of calcium and craving for food. Furthermore, in a randomized controlled trial, 55 AUD patients were given a daily dose of calcium carbonate combined with a small amount of vitamin D (5 μg) and experienced reduced alcohol craving intensity and faster attenuation of withdrawal symptoms compared to a group receiving sodium bicarbonate [28]. These results may substantiate the role of vitamin D in calcium homeostasis and the associated glutamatergic and GABAergic transmission, which could thereby affect cellular signaling. However, vitamin D also contributes to neurodevelopment, neuroprotection and the growth of dopaminergic pathways [3], and animal studies have shown the specific role of vitamin D3 in the regulation of dopamine circuits, which are involved in drug‐seeking behaviours and addictive‐like eating behaviours. Preclinical data provide evidence for dietary vitamin D3 influencing dopamine circuits, responses to drugs of abuse, intake of food and the development of obesity. For instance, in one study [6], dietary D3 deficiency in mice was reported to lead to an increased intake of amphetamine and a high‐fat diet, and an increased body weight. Taken together, our findings are concordant with these data, and the hypothesis that chronic vitamin D deficiency could be associated with reduced dopamine signaling in animals and humans leading to a ‘reward deficiency’, and thereby to a compensatory consumption of highly palatable and caloric foods or drugs [45, 46]. However, further research is warranted to investigate and validate the underlying mechanisms of this association and the respective roles of both vitamin D and calcium levels.

Due to the exploratory nature of our study, no correction for multiple comparisons was applied. Besides this issue, several limitations should be acknowledged for the present study. First, the two groups differed in sample size (232 patients vs. 28 patients), which precluded testing a Group by Time interaction on craving metrics. Second, there is no international consensus on thresholds for defining vitamin D deficiency, and various thresholds have been used across studies, thereby compromising comparisons of our results with those of previous studies. Of note, in the present study, we chose to define the optimal vitamin D status according to the French recommendations [18]. Moreover, another potential bias concerns vitamin D supplementation that was provided during hospitalization, while vitamin D concentration was not measured longitudinally. Yet, since in our study hospital discharge occurred 3 or 4 weeks after admission (with an average stay of 25 days), the effect of supplementation on vitamin D concentration is likely to have little impact, if any, on our findings, as at least a 3‐month vitamin D supplementation schedule is considered necessary to obtain a substantial increase in plasma levels [47].

Despite these caveats, our findings support further evidence for considering vitamin D plasma levels in SUD populations during addiction treatment. Given the high prevalence of poor vitamin D status among SUDs, and particularly that of severe deficiency status, the integration of vitamin D supplementation should be part of the treatment programme for these conditions. Due to the exploratory design of the present research and the extreme imbalance in sample size according to the vitamin D status, further longitudinal assessments are needed to better examine the extent to which vitamin D and calcium status prospectively predict relapse vulnerability and influence substance use outcomes and to determine the potential therapeutic effects of supplementation on craving intensity over time. Finally, the relationship between vitamin D, calcium plasma level (and their interplay) and addictive‐like eating, anthropometric and metabolic measures should also be further investigated.

## Authors Contributions

**Luca Pavirani**, **Sylvie Berthoz**, **Melina Fatseas:** conceptualization. **Pauline Desnavailles**, **Sylvie Berthoz**, **Melina Fatseas:** methodology. **Luca Pavirani**, **Bibi Aliya Seelarbokus**, **Pauline Desnavailles**, **Sylvie Berthoz**, **Melina Fatseas:** formal analysis. **Luca Pavirani**, **Bibi Aliya Seelarbokus**, **Luca Pavirani:** investigation. **Luca Pavirani**, **Bibi Aliya Seelarbokus**, **Luca Pavirani:** data curation. **Luca Pavirani**, **Sylvie Berthoz**, **Melina Fatseas:** writing – original draft preparation. **Luca Pavirani**, **Bibi Aliya Seelarbokus**, **Luca Pavirani**, **Pauline Desnavailles**, **Sylvie Berthoz**, **Melina Fatseas:** writing and editing. **Sylvie Berthoz**, **Melina Fatseas:** project administration. All authors have read and agreed to the final version of the manuscript.

## Funding

L.P. received the financial support from the scientific committee of the French annual congress of Psychiatry (Congrès Français de Psychiatrie, CFP). B.A.S. is a PhD fellow funded by the Doctoral School of Bordeaux Neurocampus Graduate Programme.

## Ethics Statement

The study was conducted in accordance with the Declaration of Helsinki, and the Institutional Review Board and local ethics committee approved the SUED study protocol (ClinicalTrials.gov NCT05315635). Written informed consent to participate was obtained from all participants prior to inclusion.

## Conflicts of Interest

The authors declare no conflicts of interest.

## Supporting information


**Data S1:** Supporting Information.

## Data Availability

The data presented in this study are available upon reasonable request from the corresponding author. The data are not publicly available due to specifications from the study sponsor.
