# Supplementary material for: Associations Between Vitamin D Status and Clinical Presentation Among French Inpatients With Substance Use Disorders
Source: Addict Biol. 2026 Jan 8;31(1):e70110. doi: 10.1111/adb.70110 (PMC12780537; doi:10.1111/adb.70110)
Supplement: Supplementary file 1 — Data S1: Supporting Information. [file ADB-31-e70110-s001.docx]

SUPPLEMENTARY RESULTS

Associations between vitamin D status and clinical presentation among French inpatients with substance use disorders

Luca Pavirani^1,2,3^, Bibi Aliya Seelarbokus^1^, Léa Marinelli^2,4^, Pauline Desnavailles^3^, Sylvie Berthoz^1,5*^, Melina Fatseas ^1,2,3^

^1^ Univ. Bordeaux, INCIA CNRS-UMR 5287, F-33000 Bordeaux, France

^2^ CHU Bordeaux, F-33000 Bordeaux, France

^3^ CH Charles Perrens, F-33000 Bordeaux, France

^4^ Univ. Bordeaux LabPsy, UR 4139, F-33000 Bordeaux, France

^5^ Department of Psychiatry for Adolescents and Young Adults, Institut Mutualiste Montsouris, F-75014 Paris, France

***Corresponding author:**Sylvie Berthoz, PhD.
INCIA CNRS UMR 5287
Bâtiment Bordeaux Biologie Santé,
2 Rue Dr Hoffmann Martinot,
33076 Bordeaux Cdx, France

sylvie.berthoz-landron@inserm.fr

**Linear Regressions with VitD level as the predictor**

**Linear Regression Craving substance 7D T1**

| Model Fit Measures | | | | |
| --- | --- | --- | --- | --- |
| **Model** | **Adjusted R²** | **AIC** | **BIC** | **RMSE** |
| 1 | -0.00632 | 463 | 470 | 2.43 |
| 2 | -0.00867 | 464 | 474 | 2.42 |
| 3 | 0.04311 | 460 | 473 | 2.34 |
| 4 | 0.04281 | 461 | 476 | 2.33 |
| *Note.* Models estimated using sample size of N=99 | | | | |

| Model Comparisons | | | | | | | |
| --- | --- | --- | --- | --- | --- | --- | --- |
| **Comparison** | | |  | | | | |
| **Model** |  | **Model** | **ΔR²** | **F** | **df1** | **df2** | **p** |
| 1 | - | 2 | 0.00797 | 0.774 | 1 | 96 | 0.381 |
| 2 | - | 3 | 0.06049 | 6.195 | 1 | 95 | 0.015 |
| 3 | - | 4 | 0.00947 | 0.970 | 1 | 94 | 0.327 |

**Model Specific Results Model 1 Model 2 Model 3 Model 4**

| Model Coefficients - Craving substance 7D T1 | | | | | | |
| --- | --- | --- | --- | --- | --- | --- |
|  | | | **95% Confidence Interval** | |  | |
| **Predictor** | **Estimate** | **SE** | **Lower** | **Upper** | **t** | **p** |
| Intercept | 2.6019 | 0.4635 | 1.6820 | 3.5218 | 5.614 | <.001 |
| Vit D (ng/ml) | -0.0156 | 0.0251 | -0.0654 | 0.0343 | -0.620 | 0.537 |

| Model Coefficients - Craving substance 7D T1 | | | | | | |
| --- | --- | --- | --- | --- | --- | --- |
|  | | | **95% Confidence Interval** | |  | |
| **Predictor** | **Estimate** | **SE** | **Lower** | **Upper** | **t** | **p** |
| Intercept | 1.6874 | 1.1383 | -0.5720 | 3.9469 | 1.482 | 0.141 |
| Vit D (ng/ml) | -0.0157 | 0.0251 | -0.0656 | 0.0341 | -0.626 | 0.533 |
| BMI | 0.0377 | 0.0429 | -0.0474 | 0.1229 | 0.880 | 0.381 |

| Model Coefficients - Craving substance 7D T1 | | | | | | |
| --- | --- | --- | --- | --- | --- | --- |
|  | | | **95% Confidence Interval** | |  | |
| **Predictor** | **Estimate** | **SE** | **Lower** | **Upper** | **t** | **p** |
| Intercept | 0.6960 | 1.1781 | -1.6427 | 3.0348 | 0.591 | 0.556 |
| Vit D (ng/ml) | -0.0140 | 0.0245 | -0.0626 | 0.0346 | -0.573 | 0.568 |
| BMI | 0.0292 | 0.0419 | -0.0540 | 0.1124 | 0.698 | 0.487 |
| HAD Anxiety T1 | 0.1313 | 0.0528 | 0.0266 | 0.2360 | 2.489 | 0.015 |

| Model Coefficients - Craving substance 7D T1 | | | | | | |
| --- | --- | --- | --- | --- | --- | --- |
|  | | | **95% Confidence Interval** | |  | |
| **Predictor** | **Estimate** | **SE** | **Lower** | **Upper** | **t** | **p** |
| Intercept | 0.8604 | 1.1900 | -1.5024 | 3.2231 | 0.723 | 0.471 |
| Vit D (ng/ml) | -0.0187 | 0.0249 | -0.0683 | 0.0308 | -0.751 | 0.455 |
| BMI | 0.0304 | 0.0419 | -0.0529 | 0.1137 | 0.725 | 0.470 |
| HAD Anxiety T1 | 0.1773 | 0.0705 | 0.0374 | 0.3172 | 2.516 | 0.014 |
| HAD Depression T1 | -0.0842 | 0.0855 | -0.2540 | 0.0856 | -0.985 | 0.327 |

**Linear Regression Craving substance T1**

| Model Fit Measures | | | | |
| --- | --- | --- | --- | --- |
| **Model** | **Adjusted R²** | **AIC** | **BIC** | **RMSE** |
| 1 | -0.00564 | 447 | 455 | 2.25 |
| 2 | -0.00430 | 448 | 458 | 2.23 |
| 3 | 0.08587 | 440 | 453 | 2.12 |
| 4 | 0.08336 | 441 | 457 | 2.11 |
| *Note.* Models estimated using sample size of N=99 | | | | |

| Model Comparisons | | | | | | | |
| --- | --- | --- | --- | --- | --- | --- | --- |
| **Comparison** | | |  | | | | |
| **Model** |  | **Model** | **ΔR²** | **F** | **df1** | **df2** | **p** |
| 1 | - | 2 | 0.01157 | 1.129 | 1 | 96 | 0.291 |
| 2 | - | 3 | 0.09765 | 10.469 | 1 | 95 | 0.002 |
| 3 | - | 4 | 0.00692 | 0.740 | 1 | 94 | 0.392 |

**Model Specific Results Model 1 Model 2 Model 3 Model 4**

| Model Coefficients - Craving substance T1 | | | | | | |
| --- | --- | --- | --- | --- | --- | --- |
|  | | | **95% Confidence Interval** | |  | |
| **Predictor** | **Estimate** | **SE** | **Lower** | **Upper** | **t** | **p** |
| Intercept | 1.6579 | 0.4289 | 0.8067 | 2.5090 | 3.866 | <.001 |
| Vit D (ng/ml) | -0.0156 | 0.0232 | -0.0617 | 0.0305 | -0.671 | 0.504 |

| Model Coefficients - Craving substance T1 | | | | | | |
| --- | --- | --- | --- | --- | --- | --- |
|  | | | **95% Confidence Interval** | |  | |
| **Predictor** | **Estimate** | **SE** | **Lower** | **Upper** | **t** | **p** |
| Intercept | 0.6378 | 1.0513 | -1.4489 | 2.7245 | 0.607 | 0.545 |
| Vit D (ng/ml) | -0.0158 | 0.0232 | -0.0618 | 0.0303 | -0.680 | 0.498 |
| BMI | 0.0421 | 0.0396 | -0.0365 | 0.1207 | 1.063 | 0.291 |

| Model Coefficients - Craving substance T1 | | | | | | |
| --- | --- | --- | --- | --- | --- | --- |
|  | | | **95% Confidence Interval** | |  | |
| **Predictor** | **Estimate** | **SE** | **Lower** | **Upper** | **t** | **p** |
| Intercept | -0.5281 | 1.0657 | -2.6438 | 1.5876 | -0.496 | 0.621 |
| Vit D (ng/ml) | -0.0138 | 0.0221 | -0.0577 | 0.0302 | -0.622 | 0.536 |
| BMI | 0.0321 | 0.0379 | -0.0432 | 0.1074 | 0.847 | 0.399 |
| HAD Anxiety T1 | 0.1544 | 0.0477 | 0.0597 | 0.2492 | 3.236 | 0.002 |

| Model Coefficients - Craving substance T1 | | | | | | |
| --- | --- | --- | --- | --- | --- | --- |
|  | | | **95% Confidence Interval** | |  | |
| **Predictor** | **Estimate** | **SE** | **Lower** | **Upper** | **t** | **p** |
| Intercept | -0.3981 | 1.0778 | -2.5381 | 1.7420 | -0.369 | 0.713 |
| Vit D (ng/ml) | -0.0175 | 0.0226 | -0.0624 | 0.0274 | -0.774 | 0.441 |
| BMI | 0.0330 | 0.0380 | -0.0424 | 0.1084 | 0.869 | 0.387 |
| HAD Anxiety T1 | 0.1908 | 0.0638 | 0.0641 | 0.3175 | 2.989 | 0.004 |
| HAD Depression T1 | -0.0666 | 0.0774 | -0.2204 | 0.0872 | -0.860 | 0.392 |

**Linear Regression Craving Food 7D T1**

| Model Fit Measures | | | | |
| --- | --- | --- | --- | --- |
| **Model** | **Adjusted R²** | **AIC** | **BIC** | **RMSE** |
| 1 | 0.00984 | 759 | 768 | 2.75 |
| 2 | 0.00387 | 761 | 773 | 2.75 |
| 3 | 0.04161 | 756 | 771 | 2.68 |
| 4 | 0.03563 | 758 | 776 | 2.68 |
| *Note.* Models estimated using sample size of N=155 | | | | |

| Model Comparisons | | | | | | | |
| --- | --- | --- | --- | --- | --- | --- | --- |
| **Comparison** | | |  | | | | |
| **Model** |  | **Model** | **ΔR²** | **F** | **df1** | **df2** | **p** |
| 1 | - | 2 | 5.44e-4 | 0.0841 | 1 | 152 | 0.772 |
| 2 | - | 3 | 0.0435 | 6.9859 | 1 | 151 | 0.009 |
| 3 | - | 4 | 3.90e-4 | 0.0623 | 1 | 150 | 0.803 |

**Model Specific Results Model 1 Model 2 Model 3 Model 4**

| Model Coefficients - Craving Food 7D T1 | | | | | | |
| --- | --- | --- | --- | --- | --- | --- |
|  | | | **95% Confidence Interval** | |  | |
| **Predictor** | **Estimate** | **SE** | **Lower** | **Upper** | **t** | **p** |
| Intercept | 3.9700 | 0.3910 | 3.1975 | 4.74249 | 10.15 | <.001 |
| Vit D (ng/ml) | -0.0317 | 0.0199 | -0.0711 | 0.00768 | -1.59 | 0.114 |

| Model Coefficients - Craving Food 7D T1 | | | | | | |
| --- | --- | --- | --- | --- | --- | --- |
|  | | | **95% Confidence Interval** | |  | |
| **Predictor** | **Estimate** | **SE** | **Lower** | **Upper** | **t** | **p** |
| Intercept | 3.6821 | 1.0674 | 1.5732 | 5.79099 | 3.450 | <.001 |
| Vit D (ng/ml) | -0.0316 | 0.0200 | -0.0711 | 0.00793 | -1.579 | 0.116 |
| BMI | 0.0117 | 0.0403 | -0.0680 | 0.09142 | 0.290 | 0.772 |

| Model Coefficients - Craving Food 7D T1 | | | | | | |
| --- | --- | --- | --- | --- | --- | --- |
|  | | | **95% Confidence Interval** | |  | |
| **Predictor** | **Estimate** | **SE** | **Lower** | **Upper** | **t** | **p** |
| Intercept | 2.57916 | 1.1271 | 0.3522 | 4.8061 | 2.288 | 0.024 |
| Vit D (ng/ml) | -0.02772 | 0.0197 | -0.0666 | 0.0112 | -1.409 | 0.161 |
| BMI | 0.00803 | 0.0396 | -0.0702 | 0.0863 | 0.203 | 0.840 |
| HAD Anxiety T1 | 0.13423 | 0.0508 | 0.0339 | 0.2346 | 2.643 | 0.009 |

| Model Coefficients - Craving Food 7D T1 | | | | | | |
| --- | --- | --- | --- | --- | --- | --- |
|  | | | **95% Confidence Interval** | |  | |
| **Predictor** | **Estimate** | **SE** | **Lower** | **Upper** | **t** | **p** |
| Intercept | 2.57907 | 1.1306 | 0.34509 | 4.8131 | 2.281 | 0.024 |
| Vit D (ng/ml) | -0.02755 | 0.0198 | -0.06658 | 0.0115 | -1.395 | 0.165 |
| BMI | 0.00678 | 0.0400 | -0.07233 | 0.0859 | 0.169 | 0.866 |
| HAD Anxiety T1 | 0.12526 | 0.0623 | 0.00209 | 0.2484 | 2.009 | 0.046 |
| HAD Depression T1 | 0.01745 | 0.0699 | -0.12068 | 0.1556 | 0.250 | 0.803 |

**Linear Regression Craving Food T1**

| Model Fit Measures | | | | |
| --- | --- | --- | --- | --- |
| **Model** | **Adjusted R²** | **AIC** | **BIC** | **RMSE** |
| 1 | 0.00661 | 770 | 779 | 2.76 |
| 2 | 0.00345 | 772 | 784 | 2.75 |
| 3 | 0.04477 | 766 | 781 | 2.69 |
| 4 | 0.03854 | 768 | 786 | 2.69 |
| *Note.* Models estimated using sample size of N=157 | | | | |

| Model Comparisons | | | | | | | |
| --- | --- | --- | --- | --- | --- | --- | --- |
| **Comparison** | | |  | | | | |
| **Model** |  | **Model** | **ΔR²** | **F** | **df1** | **df2** | **p** |
| 1 | - | 2 | 0.00325 | 0.50874 | 1 | 154 | 0.477 |
| 2 | - | 3 | 0.04691 | 7.66118 | 1 | 153 | 0.006 |
| 3 | - | 4 | 5.35e-5 | 0.00868 | 1 | 152 | 0.926 |

**Model Specific Results Model 1 Model 2 Model 3 Model 4**

| Model Coefficients - Craving Food T1 | | | | | | |
| --- | --- | --- | --- | --- | --- | --- |
|  | | | **95% Confidence Interval** | |  | |
| **Predictor** | **Estimate** | **SE** | **Lower** | **Upper** | **t** | **p** |
| Intercept | 3.7024 | 0.3875 | 2.9368 | 4.4679 | 9.55 | <.001 |
| Vit D (ng/ml) | -0.0276 | 0.0193 | -0.0658 | 0.0106 | -1.43 | 0.155 |

| Model Coefficients - Craving Food T1 | | | | | | |
| --- | --- | --- | --- | --- | --- | --- |
|  | | | **95% Confidence Interval** | |  | |
| **Predictor** | **Estimate** | **SE** | **Lower** | **Upper** | **t** | **p** |
| Intercept | 3.0129 | 1.0417 | 0.9550 | 5.0707 | 2.892 | 0.004 |
| Vit D (ng/ml) | -0.0275 | 0.0194 | -0.0657 | 0.0108 | -1.420 | 0.158 |
| BMI | 0.0281 | 0.0393 | -0.0496 | 0.1058 | 0.713 | 0.477 |

| Model Coefficients - Craving Food T1 | | | | | | |
| --- | --- | --- | --- | --- | --- | --- |
|  | | | **95% Confidence Interval** | |  | |
| **Predictor** | **Estimate** | **SE** | **Lower** | **Upper** | **t** | **p** |
| Intercept | 1.8546 | 1.1024 | -0.3232 | 4.0325 | 1.682 | 0.095 |
| Vit D (ng/ml) | -0.0255 | 0.0190 | -0.0629 | 0.0120 | -1.342 | 0.181 |
| BMI | 0.0262 | 0.0385 | -0.0499 | 0.1023 | 0.680 | 0.498 |
| HAD Anxiety T1 | 0.1384 | 0.0500 | 0.0396 | 0.2372 | 2.768 | 0.006 |

| Model Coefficients - Craving Food T1 | | | | | | |
| --- | --- | --- | --- | --- | --- | --- |
|  | | | **95% Confidence Interval** | |  | |
| **Predictor** | **Estimate** | **SE** | **Lower** | **Upper** | **t** | **p** |
| Intercept | 1.85498 | 1.1060 | -0.3301 | 4.0400 | 1.6772 | 0.096 |
| Vit D (ng/ml) | -0.02557 | 0.0191 | -0.0632 | 0.0121 | -1.3412 | 0.182 |
| BMI | 0.02670 | 0.0390 | -0.0504 | 0.1038 | 0.6842 | 0.495 |
| HAD Anxiety T1 | 0.14163 | 0.0609 | 0.0212 | 0.2620 | 2.3241 | 0.021 |
| HAD Depression T1 | -0.00647 | 0.0694 | -0.1437 | 0.1307 | -0.0932 | 0.926 |

**Linear Regressions with Calcium level as the predictor**

**Linear Regression Craving substance 7D T1**

| Model Fit Measures | | | | |
| --- | --- | --- | --- | --- |
| **Model** | **R** | **R²** | **AIC** | **BIC** |
| 1 | 0.0341 | 0.00116 | 426 | 434 |
| 2 | 0.0844 | 0.00712 | 428 | 438 |
| 3 | 0.2571 | 0.06612 | 424 | 437 |
| 4 | 0.2658 | 0.07066 | 426 | 441 |
| *Note.* Models estimated using sample size of N=91 | | | | |

| Model Comparisons | | | | | | | |
| --- | --- | --- | --- | --- | --- | --- | --- |
| **Comparison** | | |  | | | | |
| **Model** |  | **Model** | **ΔR²** | **F** | **df1** | **df2** | **p** |
| 1 | - | 2 | 0.00595 | 0.527 | 1 | 88 | 0.470 |
| 2 | - | 3 | 0.05900 | 5.497 | 1 | 87 | 0.021 |
| 3 | - | 4 | 0.00455 | 0.421 | 1 | 86 | 0.518 |

**Model Specific Results Model 1 Model 2 Model 3 Model 4**

| Model Coefficients - Craving substance D7 T1 | | | | | | |
| --- | --- | --- | --- | --- | --- | --- |
|  | | | **95% Confidence Interval** | |  | |
| **Predictor** | **Estimate** | **SE** | **Lower** | **Upper** | **t** | **p** |
| Intercept | 0.865 | 4.74 | -8.56 | 10.29 | 0.182 | 0.856 |
| Calcium mmol/L | 0.651 | 2.02 | -3.37 | 4.67 | 0.322 | 0.748 |

| Model Coefficients - Craving substance D7 T1 | | | | | | |
| --- | --- | --- | --- | --- | --- | --- |
|  | | | **95% Confidence Interval** | |  | |
| **Predictor** | **Estimate** | **SE** | **Lower** | **Upper** | **t** | **p** |
| Intercept | -0.1956 | 4.9740 | -10.0804 | 9.689 | -0.0393 | 0.969 |
| Calcium mmol/L | 0.7814 | 2.0349 | -3.2624 | 4.825 | 0.3840 | 0.702 |
| BMI | 0.0311 | 0.0428 | -0.0540 | 0.116 | 0.7263 | 0.470 |

| Model Coefficients - Craving substance D7 T1 | | | | | | |
| --- | --- | --- | --- | --- | --- | --- |
|  | | | **95% Confidence Interval** | |  | |
| **Predictor** | **Estimate** | **SE** | **Lower** | **Upper** | **t** | **p** |
| Intercept | -0.0510 | 4.8520 | -9.6948 | 9.593 | -0.0105 | 0.992 |
| Calcium mmol/L | 0.3421 | 1.9936 | -3.6204 | 4.305 | 0.1716 | 0.864 |
| BMI | 0.0198 | 0.0420 | -0.0638 | 0.103 | 0.4704 | 0.639 |
| HAD Anxiety T1 | 0.1315 | 0.0561 | 0.0200 | 0.243 | 2.3445 | 0.021 |

| Model Coefficients - Craving substance D7 T1 | | | | | | |
| --- | --- | --- | --- | --- | --- | --- |
|  | | | **95% Confidence Interval** | |  | |
| **Predictor** | **Estimate** | **SE** | **Lower** | **Upper** | **t** | **p** |
| Intercept | -0.2594 | 4.8788 | -9.9581 | 9.439 | -0.0532 | 0.958 |
| Calcium mmol/L | 0.4570 | 2.0081 | -3.5350 | 4.449 | 0.2276 | 0.820 |
| BMI | 0.0199 | 0.0422 | -0.0639 | 0.104 | 0.4724 | 0.638 |
| HAD Anxiety T1 | 0.1650 | 0.0763 | 0.0132 | 0.317 | 2.1612 | 0.033 |
| HAD Depression T1 | -0.0595 | 0.0918 | -0.2420 | 0.123 | -0.6486 | 0.518 |

**Linear Regression Craving substance T1**

| Model Fit Measures | | |
| --- | --- | --- |
| **Model** | **R** | **R²** |
| 1 | 0.0272 | 7.38e-4 |
| 2 | 0.1170 | 0.0137 |
| 3 | 0.3511 | 0.1233 |
| 4 | 0.3530 | 0.1246 |
| *Note.* Models estimated using sample size of N=91 | | |

| Model Comparisons | | | | | | | |
| --- | --- | --- | --- | --- | --- | --- | --- |
| **Comparison** | | |  | | | | |
| **Model** |  | **Model** | **ΔR²** | **F** | **df1** | **df2** | **p** |
| 1 | - | 2 | 0.01295 | 1.155 | 1 | 88 | 0.285 |
| 2 | - | 3 | 0.10958 | 10.874 | 1 | 87 | 0.001 |
| 3 | - | 4 | 0.00136 | 0.134 | 1 | 86 | 0.716 |

**Model Specific Results Model 1 Model 2 Model 3 Model 4**

| Model Coefficients - Craving substance T1 | | | | |
| --- | --- | --- | --- | --- |
| **Predictor** | **Estimate** | **SE** | **t** | **p** |
| Intercept | 0.351 | 4.47 | 0.0784 | 0.938 |
| Calcium mmol/L | 0.488 | 1.91 | 0.2563 | 0.798 |

| Model Coefficients - Craving substance T1 | | | | |
| --- | --- | --- | --- | --- |
| **Predictor** | **Estimate** | **SE** | **t** | **p** |
| Intercept | -1.3532 | 4.7386 | -0.286 | 0.776 |
| Calcium mmol/L | 0.7469 | 1.9188 | 0.389 | 0.698 |
| BMI | 0.0450 | 0.0418 | 1.075 | 0.285 |

| Model Coefficients - Craving substance T1 | | | | |
| --- | --- | --- | --- | --- |
| **Predictor** | **Estimate** | **SE** | **t** | **p** |
| Intercept | -1.0896 | 4.4940 | -0.2425 | 0.809 |
| Calcium mmol/L | 0.1566 | 1.8282 | 0.0856 | 0.932 |
| BMI | 0.0298 | 0.0399 | 0.7469 | 0.457 |
| HAD Anxiety T1 | 0.1689 | 0.0512 | 3.2975 | 0.001 |

| Model Coefficients - Craving substance T1 | | | | |
| --- | --- | --- | --- | --- |
| **Predictor** | **Estimate** | **SE** | **t** | **p** |
| Intercept | -1.2061 | 4.5277 | -0.266 | 0.791 |
| Calcium mmol/L | 0.2174 | 1.8449 | 0.118 | 0.906 |
| BMI | 0.0301 | 0.0401 | 0.751 | 0.455 |
| HAD Anxiety T1 | 0.1861 | 0.0698 | 2.668 | 0.009 |
| HAD Depression T1 | -0.0307 | 0.0840 | -0.365 | 0.716 |

**Linear Regression Craving Food D7 T1**

| Model Fit Measures | | |
| --- | --- | --- |
| **Model** | **R** | **R²** |
| 1 | 0.140 | 0.0196 |
| 2 | 0.141 | 0.0197 |
| 3 | 0.281 | 0.0788 |
| 4 | 0.285 | 0.0815 |
| *Note.* Models estimated using sample size of N=145 | | |

| Model Comparisons | | | | | | | |
| --- | --- | --- | --- | --- | --- | --- | --- |
| **Comparison** | | |  | | | | |
| **Model** |  | **Model** | **ΔR²** | **F** | **df1** | **df2** | **p** |
| 1 | - | 2 | 1.12e-4 | 0.0162 | 1 | 142 | 0.899 |
| 2 | - | 3 | 0.05906 | 9.0403 | 1 | 141 | 0.003 |
| 3 | - | 4 | 0.00267 | 0.4064 | 1 | 140 | 0.525 |

**Model Specific Results Model 1 Model 2 Model 3 Model 4**

| Model Coefficients - Craving Food D7 T1 | | | | | | |
| --- | --- | --- | --- | --- | --- | --- |
|  | | | **95% Confidence Interval** | |  | |
| **Predictor** | **Estimate** | **SE** | **Lower** | **Upper** | **t** | **p** |
| Intercept | 10.49 | 4.14 | 2.30 | 18.671 | 2.53 | 0.012 |
| Calcium mmol/L | -2.99 | 1.77 | -6.48 | 0.502 | -1.69 | 0.093 |

| Model Coefficients - Craving Food D7 T1 | | | | | | |
| --- | --- | --- | --- | --- | --- | --- |
|  | | | **95% Confidence Interval** | |  | |
| **Predictor** | **Estimate** | **SE** | **Lower** | **Upper** | **t** | **p** |
| Intercept | 10.29258 | 4.4248 | 1.5456 | 19.0396 | 2.326 | 0.021 |
| Calcium mmol/L | -2.96297 | 1.7853 | -6.4921 | 0.5662 | -1.660 | 0.099 |
| BMI | 0.00532 | 0.0419 | -0.0775 | 0.0881 | 0.127 | 0.899 |

| Model Coefficients - Craving Food D7 T1 | | | | | | |
| --- | --- | --- | --- | --- | --- | --- |
|  | | | **95% Confidence Interval** | |  | |
| **Predictor** | **Estimate** | **SE** | **Lower** | **Upper** | **t** | **p** |
| Intercept | 9.089 | 4.3232 | 0.5423 | 17.6356 | 2.1024 | 0.037 |
| Calcium mmol/L | -2.952 | 1.7368 | -6.3853 | 0.4816 | -1.6996 | 0.091 |
| BMI | -7.92e−4 | 0.0408 | -0.0814 | 0.0799 | -0.0194 | 0.985 |
| HAD Anxiety T1 | 0.159 | 0.0529 | 0.0544 | 0.2635 | 3.0067 | 0.003 |

| Model Coefficients - Craving Food D7 T1 | | | | | | |
| --- | --- | --- | --- | --- | --- | --- |
|  | | | **95% Confidence Interval** | |  | |
| **Predictor** | **Estimate** | **SE** | **Lower** | **Upper** | **t** | **p** |
| Intercept | 9.39741 | 4.3593 | 0.77892 | 18.0159 | 2.156 | 0.033 |
| Calcium mmol/L | -3.07481 | 1.7511 | -6.53684 | 0.3872 | -1.756 | 0.081 |
| BMI | -0.00436 | 0.0413 | -0.08594 | 0.0772 | -0.106 | 0.916 |
| HAD Anxiety T1 | 0.13438 | 0.0655 | 0.00481 | 0.2639 | 2.050 | 0.042 |
| HAD Depression T1 | 0.04722 | 0.0741 | -0.09921 | 0.1936 | 0.638 | 0.525 |

**Linear Regression Craving Food T1**

| Model Fit Measures | | |
| --- | --- | --- |
| **Model** | **R** | **R²** |
| 1 | 0.151 | 0.0228 |
| 2 | 0.157 | 0.0246 |
| 3 | 0.292 | 0.0852 |
| 4 | 0.293 | 0.0857 |
| *Note.* Models estimated using sample size of N=147 | | |

| Model Comparisons | | | | | | | |
| --- | --- | --- | --- | --- | --- | --- | --- |
| **Comparison** | | |  | | | | |
| **Model** |  | **Model** | **ΔR²** | **F** | **df1** | **df2** | **p** |
| 1 | - | 2 | 0.00189 | 0.2787 | 1 | 144 | 0.598 |
| 2 | - | 3 | 0.06054 | 9.4636 | 1 | 143 | 0.003 |
| 3 | - | 4 | 5.31e-4 | 0.0825 | 1 | 142 | 0.774 |

**Model Specific Results Model 1 Model 2 Model 3 Model 4**

| Model Coefficients - Craving Food T1 | | | | | | |
| --- | --- | --- | --- | --- | --- | --- |
|  | | | **95% Confidence Interval** | |  | |
| **Predictor** | **Estimate** | **SE** | **Lower** | **Upper** | **t** | **p** |
| Intercept | 10.86 | 4.13 | 2.70 | 19.023 | 2.63 | 0.009 |
| Calcium mmol/L | -3.24 | 1.76 | -6.72 | 0.245 | -1.84 | 0.068 |

| Model Coefficients - Craving Food T1 | | | | | | |
| --- | --- | --- | --- | --- | --- | --- |
|  | | | **95% Confidence Interval** | |  | |
| **Predictor** | **Estimate** | **SE** | **Lower** | **Upper** | **t** | **p** |
| Intercept | 10.0759 | 4.3988 | 1.3815 | 18.770 | 2.291 | 0.023 |
| Calcium mmol/L | -3.1265 | 1.7781 | -6.6410 | 0.388 | -1.758 | 0.081 |
| BMI | 0.0214 | 0.0406 | -0.0588 | 0.102 | 0.528 | 0.598 |

| Model Coefficients - Craving Food T1 | | | | | | |
| --- | --- | --- | --- | --- | --- | --- |
|  | | | **95% Confidence Interval** | |  | |
| **Predictor** | **Estimate** | **SE** | **Lower** | **Upper** | **t** | **p** |
| Intercept | 8.8752 | 4.2927 | 0.3899 | 17.3606 | 2.068 | 0.040 |
| Calcium mmol/L | -3.1470 | 1.7280 | -6.5628 | 0.2688 | -1.821 | 0.071 |
| BMI | 0.0177 | 0.0395 | -0.0603 | 0.0958 | 0.450 | 0.654 |
| HAD Anxiety T1 | 0.1596 | 0.0519 | 0.0570 | 0.2621 | 3.076 | 0.003 |

| Model Coefficients - Craving Food T1 | | | | | | |
| --- | --- | --- | --- | --- | --- | --- |
|  | | | **95% Confidence Interval** | |  | |
| **Predictor** | **Estimate** | **SE** | **Lower** | **Upper** | **t** | **p** |
| Intercept | 9.0078 | 4.3312 | 0.4458 | 17.5698 | 2.080 | 0.039 |
| Calcium mmol/L | -3.1991 | 1.7431 | -6.6448 | 0.2466 | -1.835 | 0.069 |
| BMI | 0.0160 | 0.0401 | -0.0632 | 0.0952 | 0.399 | 0.690 |
| HAD Anxiety T1 | 0.1490 | 0.0637 | 0.0230 | 0.2750 | 2.338 | 0.021 |
| HAD Depression T1 | 0.0210 | 0.0731 | -0.1236 | 0.1656 | 0.287 | 0.774 |
